# Preventive effect of flavonoids from Wushan Shencha (*Malus doumeri* leaves) on CCl_4_‐induced liver injury

**DOI:** 10.1002/fsn3.1243

**Published:** 2019-10-15

**Authors:** Kai Zhu, Guangbin Huang, Jing Xie, Xianrong Zhou, Jianfei Mu, Xin Zhao

**Affiliations:** ^1^ Chongqing Collaborative Innovation Center for Functional Food Chongqing University of Education Chongqing China; ^2^ Chongqing Engineering Research Center of Functional Food Chongqing University of Education Chongqing China; ^3^ Chongqing Engineering Laboratory for Research and Development of Functional Food Chongqing University of Education Chongqing China; ^4^ Department of Trauma Surgery, Emergency Medical Center of Chongqing the Affiliated Central Hospital of Chongqing University Chongqing China

**Keywords:** flavonoid, liver injury, mRNA, Wushan Shencha

## Abstract

Wushan Shencha (*Malus doumeri* leaf) is a unique tea‐like drink. Herein, the effect of flavonoids from Wushan Shencha (FWSSC) on carbon tetrachloride‐induced liver injury was studied. The serum and liver tissues of experimental mice were analyzed by kits, a slice technique, and qPCR assay. The liver index is a calculated liver‐to‐body weight ratio, and the experimental results showed that FWSSC reduced the liver index of the model group with liver injury, which was the highest. Sections stained with H&E showed that FWSSC reduced stem cell necrosis caused by liver injury. FWSSC reduced the serum levels of AST, ALT, TG, and TC, as well as the levels of IL‐6, TNF‐α, and IFN‐γ cytokines in the serum of mice with liver injury. Liver biochemical tests also showed that FWSSC increased the SOD activity and decreased TC, TG, and MPO levels in mice with liver injury. It was found that FWSSC upregulated the expression of Cu/Zn‐SOD, Mn‐SOD, CAT, and IκB‐α, and downregulated the expression of NF‐κB, COX‐2, TNF‐α, and IL‐1β in the liver tissue of mice with liver injury by detecting the expression of mRNA in liver tissue. It is concluded that FWSSC is an active substance with hepatoprotective effects. The activity of FWSSC increased with increasing concentration, and the hepatoprotective effect of FWSSC at 100 mg/kg concentration was stronger than that of silymarin.

## 1 INTRODUCTION

Wushan Shencha comes from the Three Gorges Valley of the Middle Yangtze River in China. The leaves of the Taiwan crabapple (*Malus doumeri*) are delicately processed to make the drink Wushan Shencha, which is often used to relieve summer heat (Yi, Wu, Tan, Li, & Zhao, 2014). Grown in a natural pollution‐free environment, the pure wild tea is rich in minerals such as nitrogen, phosphorus, potassium, calcium, magnesium, zinc, and manganese, as well as a variety of vitamins, amino acids, and flavonoids. It has anticancer, antioxidation, antiaging, antimildew, bacteriostasis, immunity enhancement, and other active functions (Sharangi, 2009).

The liver is the body's detoxification organ and is the largest digestive organ with the greatest participation in metabolic functions of all the organs. It participates in thousands of chemical reactions in the body, and if the liver is severely damaged and the function is abnormal, it will have a severe impact on the body. With increased economic growth, people's dietary habits have also changed, and there has been a steady increase in fatty liver, alcoholic liver, drug‐damaged liver, and other liver diseases that is closely related to numerous factors such as a high‐fat diet, excessive alcohol consumption, indiscriminate use of medication, drug abuse, smoking, and lack of sleep, which will cause liver damage (Lee et al., 2007).

The pathological basis of chemical liver injury is lipid peroxidation and free radical production. As an intermediate product of energy transfer, free radicals participate in various physiological and biochemical reactions in the human body. The dynamic balance of the amount of free radicals is of great significance for maintaining the stability and health of the human internal environment. When stimulated by external environmental factors or aging, the accumulation rate of free radicals in cells is faster than that of scavenging, resulting in oxidative stress, which is manifested by damage to cells, tissues, and organs as well as loss of function, chromosomal variation, and even death (Weber, Boll, & Stampfl, 2003).

Carbon tetrachloride (CCl_4_) is often administered to laboratory rats and mice to create an animal model of liver injury. The main mechanism of injury is driven by the free radical metabolites of CCl_4_, whereby CCl_4_ produces toxic metabolites through cytochrome P4502El metabolism in the liver (Manibusan, Odin, & Eastmond, 2007). The lysozyme effect of CCl_4_ itself could lead to hepatocyte damage, although the process of hepatic damage caused by free radicals of CCl_4_ is considered to be the main mechanism (Lettéron et al., 1990). Reactive oxygen species (ROS)‐induced oxidative stress causes a certain degree of damage to hepatocytes, which may play a key role in the pathogenesis of various liver injuries. ROS mainly comes from the process of ATP production by electron transfer in the mitochondrial respiratory chain complex. The mitochondria are abundant in the liver, and thus, the liver becomes the main organ that is attacked by ROS. The resulting oxidative stress can cause acute liver injury and negatively affect the body (Pramyothin, Janthasoot, Pongnimitprasert, Phrukudom, & Ruangrungsi, 2004).

Flavonoids have a wide range of functions and strong antioxidant activity (van Acker et al., 1996). By eliminating a great number of oxygen free radicals from the body, they can slow down the aging and degeneration of cells to prevent the occurrence of cancer (Trueba, Sánchez, & Giuliani, 2004). Flavonoids promote blood circulation, reduce cholesterol, and ameliorate cardiovascular and cerebrovascular diseases or reduce their incidence (Knekt et al., 2000, 2002). Flavonoids also promote wound healing by inhibiting the exudation of inflammatory enzymes. Some studies have found that the flavonoids in different plants have preventive effects on carbon tetrachloride‐induced liver injury (Xie et al., 2018; Zhang et al., 2012; Yuan et al., 2008). However, the preventive effect of flavonoids from Wushan Shencha on carbon tetrachloride‐induced liver injury has not yet been reported.

In this study, the flavonoids from Wushan Shencha were extracted, and their preventive effect on acute liver injury in mice was tested by establishing a CCl_4_‐induced liver injury model. The serum and liver tissues of mice were tested, and biochemical and molecular biology experiments showed that the flavonoids of Wushan Shencha had a good preventive effect on experimental acute liver injury. These experimental results will provide the theoretical basis for further utilization of this resource.

## 2 MATERIALS AND METHODS

### 2.1 Extraction of flavonoids

A mixture was created of 1.3 g of Na_2_CO_3_, 2.6 g of Na_2_B_4_O_7_, and 60 g of crushed Wushan Shencha leaves, which was then extracted by adding 600 ml of 40% ethanol solution and incubating at 50°C for 30 min. The solution was centrifuged, the supernatant was removed, and 600 ml of 40% ethanol solution was added to the precipitate, which was incubated at 50°C for 30 min. The supernatant was separated by centrifugation. After two extractions, the supernatant was adjusted to pH 4.0 with 12% hydrochloric acid, and then, the flavonoid extract was obtained by rotary evaporation (R‐1001‐VN; Zhengzhou Greatwall Scientific Industrial and Trade Co., Ltd.) (Qian et al., 2018).

### 2.2 Determination of the purity of flavonoids

A rutin standard solution (10, 20, 30, 40, and 50 μg/ml, Shanghai Yuanye Biotechnology Co., Ltd.) was prepared by adding 90% (volume ratio) ethanol solution. Then, the crude extract of Wushan Shencha flavonoids was also dissolved in ethanol solution with a volume ratio of 90%. The absorbance of the Wushan Shencha flavonoid solution was determined at 500 nm. The content of total flavonoids (by rutin) was calculated according to the standard curve of a rutin standard solution (Wang et al., 2019).

### 2.3 Animal experiments

Fifty 6‐week‐old SPF Kunming mice (male, weight of 20 ± 2 g, Experimental Animal Center of Chongqing Medical University) were fed with standard laboratory diet, and pads to absorb waste in the cages were changed every 2 days. After adaptive feeding for a week, the mice were divided into five groups: normal group, model group, FWSSCL group, FWSSCH group, and silymarin group, with 10 mice for each group. Normal and model mice were fed with gastric perfusion. FWSSCL and FWSSCH mice were fed 50 and 100 mg/kg of flavonoids, respectively, for 14 days. The silymarin mouse group was fed with silymarin at a concentration of 100 mg/kg for 14 days. On the 14th day, all mice except those in the normal group were injected with CCl_4_ inducer (the volume ratio of CCl_4_ to olive oil was 1:1, 0.1 ml/10 g) (Liu et al., 2019). After intraperitoneal injection of CCl_4_ solution, all experimental mice underwent fasting for 24 hr and were euthanized, and the liver and blood were then taken for use. Additionally, the liver tissue index (%) = liver mass (g)/ body mass of mice (kg) × 100 was determined. Diet and drinking of mice water were recorded. This study was approved by the Animal Ethics Committee of Chongqing University of Education (201903001B).

### 2.4 Serum levels in mice

After mouse plasma centrifugation at 730 × g for 10 min, the upper serum was collected, and the levels of aspartate aminotransferase/transaminase (AST, C010‐2‐1), alanine aminotransferase (ALT, C009‐2‐1), triglyceride (TG, A110‐2‐1), and total cholesterol (TC, A111‐2‐1) were determined according to the kit instructions (Nanjing Jiancheng Bioengineering Institute).

### 2.5 Liver tissue levels in mice

The livers from the mice were processed into 10% homogenates and centrifuged at 730 × g for 10 min. The supernatant was removed, and the levels of TG, TC, superoxide dismutase (SOD, A001‐3‐2), and myeloperoxidase (MPO, A044‐1‐1) in the liver tissue were determined according to the kit instructions (Nanjing Jiancheng Bioengineering Institute).

### 2.6 Pathological observation

The liver tissue was removed from the fixative, and the target tissue was smoothed. The trimmed tissues and corresponding labels are dehydrated in the dehydration box, and the wax‐soaked tissues were embedded in the embedding machine (Histocentre 3, Thermo Fisher Scientific). The embedded slices were cooled on −20°C freezing table, and the wax was solidified; then, the wax blocks were removed and repaired. Then, the wax blocks were placed on the paraffin slicer (Finesse E+, Thermo Fisher Scientific) and sliced into the 60°C oven to bake the slices. Sections were baked and dried, and then were stored at room temperature. The sections were put into xylene for 20 min, absolute ethanol for 10 min, then into ethanol with volume fractions of 95%, 90%, 80%, and 70%, respectively, for 5 min, and then washed with distilled water. The sections were dyed in hematoxylin dye solution (H&E, ab245880; Abcam) for 8 min, washed with distilled water, and then differentiated with 1% hydrochloric acid and alcohol for 10 s. The sections were washed with distilled water, then returned to blue with 0.6% ammonia water, and rinsed with running water. The sections were dyed in eosin dye for 3 min, then dehydrated, dried, and sealed with neutral gum. The morphological changes in the liver tissues were observed under an optical microscope (BX43; Olympus) and analyzed by drug‐induced liver injury (DILI) method. Hepatocyte steatosis score was 3 (bullous 1, vesicular 2), hepatocellular cholestasis score was 1, apoptotic body score was 1, eosinophilic leukocyte infiltration score was 2, above total score was 7; epithelial granuloma score was 1, and iron deposition score was 1 in necrotic area.

### 2.7 Quantitative PCR (qPCR) assay

The liver tissue of mice was crushed, and then, the general RNA in tongue tissue was extracted by RNAzol (Invitrogen). The RNA concentration was diluted to 1 μg/μl. Then, a diluted general RNA solution of 5 μL was extracted and retrieved by a reverse transcription kit to obtain the cDNA template. Next, 2 μL of cDNA template was mixed with 10 μL of SYBR Green PCR Master Mix and 1 μL upstream and downstream primers (Table 1, Thermo Fisher Scientific), and underwent PCR at 95°C for 15 s, 55°C for 30 s, and then 40 cycles at 72°C for 35 s. Finally, the relative gene expression was calculated by the 2^‐ΔΔ^ *^C^* ^t^ method with β‐actin as the internal reference at 95°C for 30 s and 55°C for 35 s (StepOnePlus Real‐Time PCR System; Thermo Fisher Scientific) (Zhang et al., 2018).

**Table 1:** Sequences of primers used in the qPCR assay

Gene name	Sequences
Cu/Zn‐SOD	Forward: 5′‐AACCAGTTGTGTTGTCAGGAC‐3′
	Reverse: 5′‐CCACCATGTTTCTTAGAGTGAGG‐3′
Mn‐SOD	Forward: 5′‐CAGACCTGCCTTACGACTATGG‐3′
	Reverse: 5′‐CTCGGTGGCGTTGAGATTGTT‐3′
CAT	Forward: 5′‐GGAGGCGGGAACCCAATAG‐3′
	Reverse: 5′‐GTGTGCCATCTCGTCAGTGAA‐3′
NF‐κB	Forward: 5′‐ATGGCAGACGATGATCCCTAC‐3′
	Reverse: 5′‐CGGAATCGAAATCCCCTCTGTT‐3′
IκB‐α	Forward: 5′‐TGAAGGACGAGGAGTACGAGC‐3′
	Reverse: 5′‐TGCAGGAACGAGTCTCCGT‐3′
COX−2	Forward: 5′‐GGTGCCTGGTCTGATGATG‐3′
	Reverse: 5′‐TGCTGGTTTGGAATAGTTGCT‐3′
TNF‐α	Forward: 5′‐GACCCTCAGACTCAGATCATCCTTCT‐3′
	Reverse: 5′‐ACGCTGGCTCAGCCACTC‐3′
IL−1β	Forward: 5′‐CTCCATGAGCTTTGTACAAGG‐3′
	Reverse: 5′‐TGCTGATGTACCAGTTGGGG‐3′
β‐actin	Forward: 5′‐AACTCCATCATGAAGTGTGA‐3′
	Reverse: 5′‐ACTCCTGCTTGCTGATCGAC‐3′

### 2.8 Western blot

The 100 microgram liver tissues were homogenized (2,190 × g for 5 min at 4°C, Hangzhou Allsheng Instrument Co Ltd.) with 1 ml RIPA and 10 L PMSF (Thermo Fisher Scientific). After the intermediate protein layer solution was removed, the protein was quantified by BCA protein quantitative kit (Beijing Solarbio Technology Co. Ltd.). The protein (50 g/ml) was mixed with the sample buffer (Thermo Fisher Scientific) at 4:1, then heated for 5 min at 100°C, and ice bath for 5 min. Then, mixing acrylamide, resolving buffer, stacking buffer, distilled water, 10% APS, and TEMED (Thermo Fisher Scientific) were mixed to make SDS‐PAGE separating and concentrating glue and poured into the running rubber board. Prestained Protein Ladder and samples were put into the sample hole of the rubber sheet, respectively, and then, the SDS‐PAGE glue containing protein was subjected to 50 min vertical gel electrophoresis. PVDF membranes (Thermo Fisher Scientific) were activated by methanol for 1 min and then transmembrane. After transmembrane was completed, PVDF membranes were sealed for 1 hr by 1 × TBST (Beijing Solarbio Technology Co. Ltd.) solution containing 5% skim milk (Becton, Dickinson and Company). After closure, the PVDF membrane was cleaned by 1 × TBST, and the first antibody was incubated at 25°C for 2 hr; the second antibody was incubated at 25°C after 5 times of cleaning by 1 × TBST. Finally, Supersignal West Pico PLUS (Thermo Fisher Science) was used to spray PVDF film and placed in iBright FL1000 (Thermo Fisher Science) for observation (Zhang et al., 2018).

### 2.9 Statistical analysis

Three parallel experiments were carried out on the serum and tissue indexes of each mouse, and then, the average values were taken. The data were analyzed by SAS 9.1 statistical software. The one‐way analysis of variance (ANOVA) method was used to determine whether there were significant differences among the groups at the level of *p* < .05.

## 3 RESULTS

### 3.1 Purity of flavonoids

**Figure 1:**
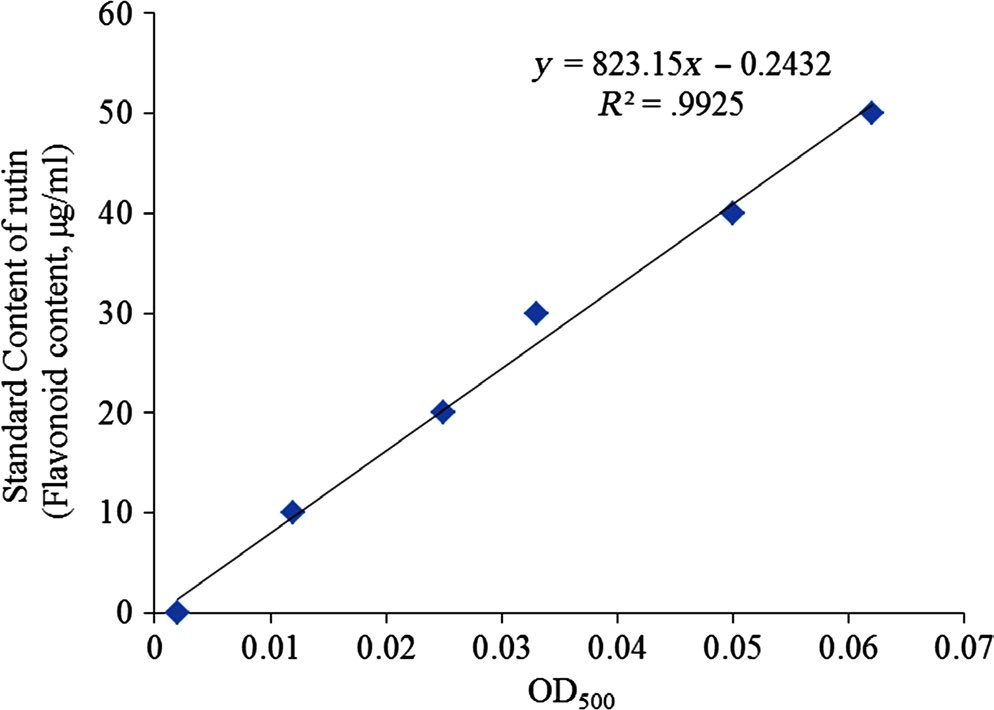
Standard curve of flavonoids (rutin)

The experimental results showed that the regression equation of the standard curve of rutin standard solution is *Y* = 823.15*X*−0.2432 (*R* ^2^ = .9925, Figure 1). Y indicates the concentration of rutin, and *X* indicates the absorbance value. According to the standard curve calculation, the content of flavonoids (rutin) in Wushan Shencha was 66.3%, which indicated that the main component in the follow‐up animal experiments was the flavonoids of Wushan Shencha.

### 3.2 Diet and drinking water of mice

**Figure 2:**
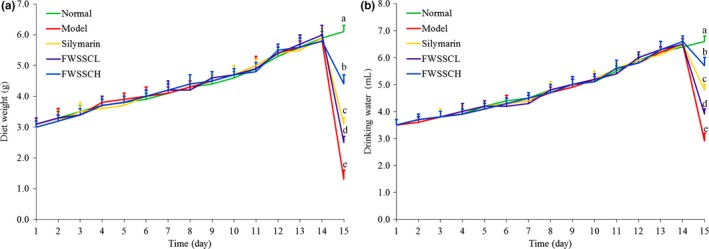
Diet (a) and drinking water (b) of mice. ^a−e^Mean values with different letters in the bar are significantly different (*p* < .05) according to Duncan's new MRT. Silymarin group: 100 mg/kg b.w. silymarin treatment dose; FWSSCL group: 50 mg/kg b.w. Wushan Shencha flavonoids dose; FWSSCH group: 100 mg/kg b.w. Wushan Shencha flavonoids dose

As can be seen from Figure 2, the mice in each group had normal diet and drinking water in the first 14 days, and there was no significant difference. After carbon tetrachloride was induced on the 14th day, the diet and drinking water of mice in each group decreased significantly (*p* > .05) compared with the normal group, the model group decreased the most, and the FWSSCH group decreased the least. It can be seen that FWSSC had no effect on the diet and drinking water of mice before inducing liver injury. After inducing liver injury, FWSSC could alleviate the abnormality of mice caused by carbon tetrachloride and alleviate the drastic decrease in mice's intake of diet and drinking water.

### 3.3 Liver index

As shown in Table 2, the flavonoids of Wushan Shencha reduced the liver weight and liver index of mice with liver injury. The liver weight and liver index of the low‐concentration (FWSSCL) and high‐concentration (FWSSCH) groups were significantly lower than those of the control group. The liver weight and liver index of the positive control (silymarin) group were lower than those of the control group. The liver weight and liver index of mice with liver injury were decreased in the high‐concentration group, and the effect was similar to that of the positive control group.

**Table 2:** Liver weight and liver index of experimental mice

Group	Body weight (g)	Liver weight (g)	Liver index (%)
Normal	41.354 ± 0.943^a^	1.902 ± 0.106^b^	4.602 ± 0.314^b^
Model	38.368 ± 1.804^a^	2.525 ± 0.273^a^	6.591 ± 0.767^a^
Silymarin	37.424 ± 1.238^b^	1.893 ± 0.131^b^	5.058 ± 0.306^ab^
FWSSCL	38.200 ± 1.582^a^	1.938 ± 0.182^b^	5.066 ± 0.315^ab^
FWSSCH	38.126 ± 0.938^b^	1.903 ± 0.265^b^	4.983 ± 0.604^ab^

Notes

Values presented are the mean ± standard deviation. ^a−e^Mean values with different letters in the same column are significantly different (*p* < .05) according to Duncan's new MRT. Silymarin group: 100 mg/kg b.w. silymarin treatment dose; FWSSCL group: 50 mg/kg b.w. Wushan Shencha flavonoids dose; FWSSCH group: 100 mg/kg b.w. Wushan Shencha flavonoids dose.

Liver injury not only threatens the health of the body, but it also can endanger life in severe cases. Carbon tetrachloride induces liver injury, mainly by the action of liver microsomal enzymes that decompose carbon tetrachloride into trichloromethane free radicals, resulting in lipid peroxidation. Carbon tetrachloride‐induced liver injury is a classical animal model of chemical liver injury. The degree of liver injury induced by carbon tetrachloride is evaluated by liver quality and liver index, and has been used in research. A high liver quality and liver index is one of the manifestations of liver injury (Wang et al., 2009). The results obtained in this study are similar to those obtained in mice with liver injury. Compared with mice with liver injury, the flavonoids of Wushan Shencha decrease the liver quality and liver index, and the effect is close to that of silymarin.

### 3.4 AST, ALT, TG, and TC levels in mice

Table 3 shows that the serum levels of AST, ALT, TG, and TC in normal mice were the lowest, but those in the model mice were the highest. The levels of AST, ALT, TG, and TC in mice with liver injury decreased after treatment with flavonoids from Wushan Shencha, and the effect was more pronounced with increasing concentration. There was no significant difference between the effect of flavonoids from Wushan Shencha and silymarin at 100 mg/kg concentration (*p* > .05).

**Table 3:** AST, ALT, TG, and TC levels in serum of experimental mice

Groups	AST (U/L)	ALT (U/L)	TG (µmol/dl)	TC (mg/dl)
Normal	16.328 ± 1.151^e^	26.558 ± 2.351^e^	30.268 ± 1.276^e^	83.652 ± 3.405^b^
Model	72.568 ± 4.337^a^	178.962 ± 9.637^a^	65.178 ± 4.336^a^	112.272 ± 6.725^d^
Silymarin	28.932 ± 1.886^c^	61.523 ± 3.185^c^	46.373 ± 1.520^c^	95.326 ± 3.873^b^
FWSSCL	47.605 ± 2.391^b^	105.789 ± 5.336^b^	55.077 ± 2.308^b^	106.320 ± 2.074^c^
FWSSCH	23.871 ± 1.792^d^	49.832 ± 2.610^d^	41.058 ± 1.294^d^	91.249 ± 2.911^b^

Notes

Values presented are the mean ± standard deviation. ^a−e^Mean values with different letters in the same column are significantly different (*p* < .05) according to Duncan's new MRT. Silymarin group: 100 mg/kg b.w. silymarin treatment dose; FWSSCL group: 50 mg/kg b.w. Wushan Shencha flavonoids dose; FWSSCH group: 100 mg/kg b.w. Wushan Shencha flavonoids dose.

The enzymes AST and ALT are the most sensitive indicators for the diagnosis of hepatocyte damage. Under normal conditions, the blood levels of ALT and AST are very low, and therefore, the activities of the two enzymes in normal serum are very low. When the liver tissue is damaged and the permeability of the cell membrane increases, these two enzymes infiltrate into the blood. The activity of these enzymes in serum then significantly increases, and thus, the increase in AST and ALT in serum reflects the degree of damage of hepatocytes and the extent of liver injury (Dong et al., 2013). The experimental data in this study also confirm that CCl_4_ increases AST and ALT levels in mice. The flavonoids of Wushan Shencha reduce the levels of AST and ALT in the serum of mice, thus playing a role in preventing liver injury.

### 3.5 Serum cytokine interleukin (IL)‐6, tumor necrosis factor (TNF)‐α, and interferon (IFN)‐γ levels in mice

Table 4 shows that the serum levels of IL‐6, TNF‐α, and IFN‐γ cytokines in normal mice were the lowest, while the levels of IL‐6, TNF‐α, and IFN‐γ cytokines in mice with CCl_4_‐induced liver injury were significantly increased. Wushan Shencha flavonoids and silymarin significantly inhibited the increase in cytokine levels caused by liver injury (*p* > .05). The levels of IL‐6, TNF‐α, and IFN‐γ cytokines in the silymarin group were close to those in the FWSSCH group, and the levels of cytokines in the FWSSCL group were only lower than those in the model group.

**Table 4:** IL‐6, TNF‐α and IFN‐γ levels in serum of experimental mice

Groups	IL−6 (pg/ml)	TNF‐α (pg/ml)	IFN‐γ ( pg/ml)
Normal	42.572 ± 2.361^e^	35.292 ± 1.496^e^	25.598 ± 1.520^e^
Model	159.742 ± 6.833^a^	133.08 ± 5.255^a^	114.706 ± 4.856^a^
Silymarin	61.259 ± 4.519^c^	59.784 ± 3.510^c^	55.108 ± 3.287^c^
FWSSCL	89.785 ± 3.974^b^	82.399 ± 4.082^b^	86.058 ± 3.874^b^
FWSSCH	50.878 ± 3.526^d^	45.773 ± 2.518^d^	38.719 ± 0.992^d^

Notes

Values presented are the mean ± standard deviation. ^a−e^Mean values with different letters in the same column are significantly different (*p* < .05) according to Duncan's new MRT. Silymarin group: 100 mg/kg b.w. silymarin treatment dose; FWSSCL group: 50 mg/kg b.w. Wushan Shencha flavonoids dose; FWSSCH group: 100 mg/kg b.w. Wushan Shencha flavonoids dose.

TNF‐α is a polypeptide mediator with extensive biological activity, and it mediates liver injury caused by various reasons. The increase in TNF‐α is directly related to liver injury (Zhao, Qian, Li, & Tan, 2015). IL‐6 stimulates hepatocytes to synthesize acute phase proteins and participate in inflammation. IL‐6 also effectively promotes cachexia induced by TNF and IL‐1 and aggravates tissue damage (Luckey & Petersen, 2001). IFN‐γ mediates the antiviral effect of nontarget cell injury, and TNF‐α participates in the injury response, promoting liver injury and inflammation of liver tissue (Shim et al., 2009). This study also confirmed that the flavonoids from Wushan Shencha prevent and alleviate liver injury by reducing IL‐6, TNF‐α, and IFN‐γ cytokines in mice.

### 3.6 Liver tissue TG, TC, SOD, and MPO levels in mice

Table 5 shows that the SOD activity in the liver tissue of the model group was the lowest, while the levels of TG, TC, and MPO were the highest. The liver tissue of normal mice showed the opposite situation, where SOD activity in liver tissue was the highest, and the levels of TG, TC, and MPO were the lowest. The flavonoids of Wushan Shencha altered the SOD activity and the levels of TG, TC, and MPO in the liver tissue of mice with liver injury so that they were almost the same as that of the normal group, and the higher the concentration of the flavonoids, the more obvious the effect. The higher the concentration of Wushan Shencha flavonoids, the more similar the observed effect is to that of silymarin.

**Table 5:** TC, TG, SOD, and MPO levels in the hepatic tissue of experimental mice

Groups	SOD (U/mg prot)	TC (μmol/g)	TG (mg/g)	MPO (μg/ml)
Normal	80.392 ± 1.209^a^	13.229 ± 1.246^e^	9.726 ± 1.026^e^	10.325 ± 0.516^d^
Model	50.392 ± 1.521^e^	24.630 ± 1.301^a^	18.921 ± 1.628^a^	15.122 ± 1.692^a^
Silymarin	63.022 ± 1.926^c^	18.211 ± 1.108^c^	13.578 ± 1.288^c^	12.159 ± 0.909^b^
FWSSCL	57.639 ± 2.012^d^	20.005 ± 1.517^b^	15.779 ± 1.302^b^	13.569 ± 0.438^c^
FWSSCH	69.283 ± 2.241^b^	16.201 ± 1.336^d^	11.113 ± 1.316^d^	11.836 ± 0.623^b^

Notes

Values presented are the mean ± standard deviation. ^a−e^Mean values with different letters in the same column are significantly different (*p* < .05) according to Duncan's new MRT. Silymarin group: 100 mg/kg b.w. silymarin treatment dose; FWSSCL group: 50 mg/kg b.w. Wushan Shencha flavonoids dose; FWSSCH group: 100 mg/kg b.w. Wushan Shencha flavonoids dose.

Carbon tetrachloride poisoning can damage the endoplasmic reticulum, resulting in a significant reduction in protein synthesis, while the TG generated in liver tissue is excreted in the form of very low density lipoprotein (VLDL)‐TG. When apolipoprotein synthesis is insufficient, VLDL is insufficient to transport TG. As a result, TC will also accumulate in the liver (Pan, Long, Yi, & Zhao, 2018). This study shows that the amount of TG and TC in the liver tissue of the model group significantly increased, while the amount of TC in the liver tissue of the model group significantly increased. The decrease in flavonoids in Wushan Shencha and silymarin showed that the flavonoids in Wushan Shencha could alleviate CCl_4_‐induced liver injury.

SOD is an antioxidant enzyme in vivo. It can eliminate harmful substances produced via metabolic processes and scavenge free radicals. The activity of SOD is proportional to the speed of scavenging free radicals. This study shows that flavonoids of Wushan Shencha can enhance the activity of SOD. The main function of MPO is to kill microorganisms in phagocytic cells, produce hypochlorite from hydrogen peroxide and chloride ions, and produce free radicals with oxidative capacity. When the defense reaction exceeds that of local antioxidants, it will lead to oxidative tissue damage (Araújo Júnior et al., 2016). MPO is closely related to inflammation. MPO‐deficient neutrophils cause oxidative reaction and produce a large number of superoxide and oxide, resulting in cell damage and inflammation (Ohta, Kongo, Sasaki, Nishida, & Ishiguro, 1999). The results showed that the MPO activity of the control group was the highest, while the MPO activity significantly decreased after treatment with Wushan Shencha flavonoids, indicating that Wushan Shencha flavonoids had the effect of repairing oxidative damage, thus playing a role in preventing liver injury.

### 3.7 Pathological observation of liver tissue

**Figure 3:**
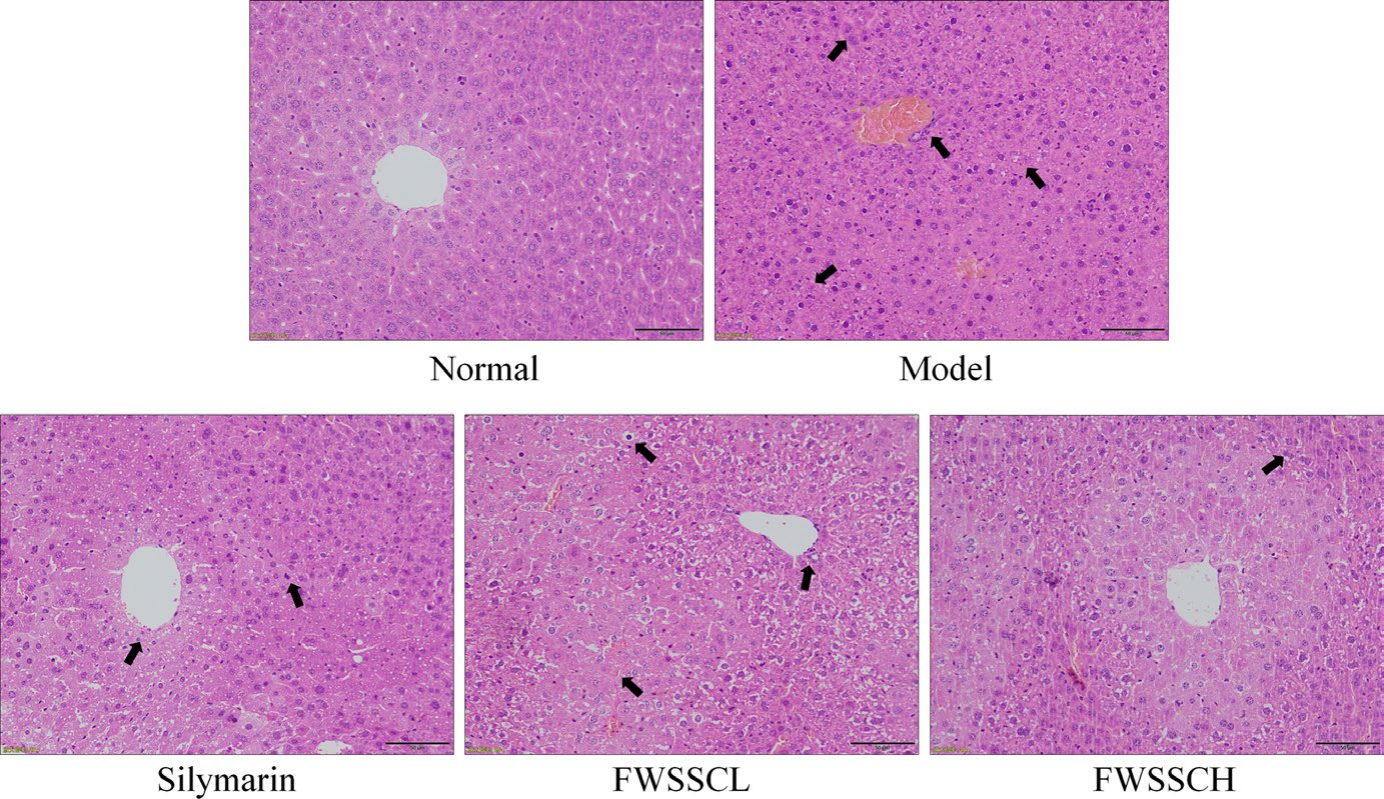
Pathological observation of hepatic injury in mice with liver injury. Silymarin group: 100 mg/kg b.w. silymarin treatment dose; FWSSCL group: 50 mg/kg b.w. Wushan Shencha flavonoids dose; FWSSCH group: 100 mg/kg b.w. Wushan Shencha flavonoids dose

Figure 3 shows that the structure of hepatic lobules in control mice is normal. The hepatocytes radiate in a normal manner from the central vein, and there is no degeneration or necrosis of hepatocytes and no inflammatory cell infiltration in the portal area. In the model group, the hepatocytes showed diffuse edema and steatosis, and the hepatocytes around the central vein showed massive necrosis (DILI 6 points). Compared with the low‐concentration FWSSCL (DILI 4 points), the necrosis area of the high‐concentration FWSSCH group was smaller, but some hepatocytes were enlarged. In the FWSSCH group (DILI 2 points) and silymarin group (DILI 3 points), the liver tissue structure was normal, but hepatocyte edema was still observed. Most of the hepatocytes did not show obvious necrosis, and only a few of them showed punctate necrosis. However, the degree of edema was significantly less than that of the control group, and no balloon‐like changes were observed. The degree of hepatocyte necrosis was also significantly reduced.

### 3.8 Cu/Zn‐SOD, Mn‐SOD, and catalase (CAT) mRNA expression in the liver tissue of mice

**Figure 4:**
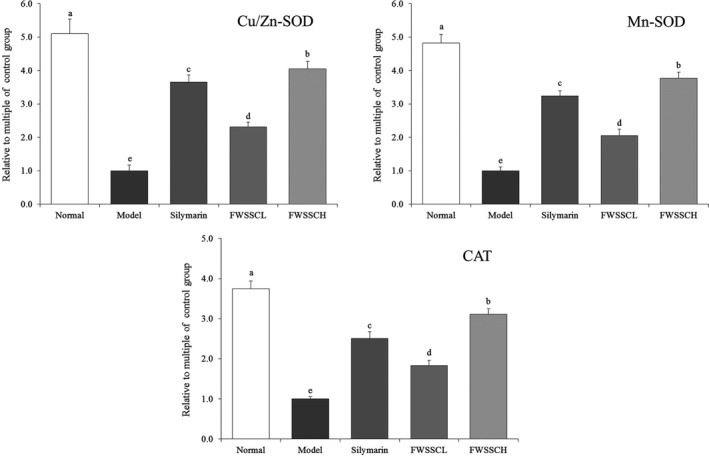
The mRNA expression of Cu/Zn‐SOD, Mn‐SOD, and CAT in the liver tissue of mice. Values presented are the mean ± standard deviation. ^a−e^Mean values with different letters in the bar are significantly different (*p* < .05) according to Duncan's new MRT. Silymarin group: 100 mg/kg b.w. silymarin treatment dose; FWSSCL group: 50 mg/kg b.w. Wushan Shencha flavonoids dose; FWSSCH group: 100 mg/kg b.w. Wushan Shencha flavonoids dose

Figure 4 shows that the expression intensity of Cu/Zn‐SOD, Mn‐SOD, and CAT in the model group was the lowest. The expression of Cu/Zn‐SOD, Mn‐SOD, and CAT in the liver tissues of mice with liver injury was significantly increased after the action of Wushan Shencha flavonoids (*p* < .05). The effect of FWSSCH was stronger than that of FWSSCL, and the effect was close to that of silymarin.

Cu/Zn‐SOD and Mn‐SOD are isomers of SOD in organisms. Mn‐SOD is a free radical scavenger, with Mn^4+^ as the active center in mitochondria. The liver contains abundant mitochondria, and after carbon tetrachloride‐induced liver injury, the activity of Mn‐SOD significantly decreases. The results of this study are consistent with this. Cu/Zn‐SOD is a free radical scavenger of SOD with Cu^2+^ and Zn^2+^ as the active centers in the cytoplasm to protect visceral tissues [30]. Studies have shown that carbon tetrachloride induces oxidative stress in organisms and produce a large number of free radicals. Mn‐SOD and Cu/Zn‐SOD inhibit free radicals and prevent liver injury (Sipes, Sisi, Sim, Mobley, & Earnest, 1991; Bonthius, Winters, Karacay, Bousquet, & Bonthius, 2015). CAT is an antioxidant enzyme that inhibits oxidative stress by clearing the body of hydrogen peroxide, slowing the oxidation caused by carbon tetrachloride, and thus inhibiting liver injury (Lee, Hyun, Jenner, & Halliwell, 2001). After liver injury, reactive oxygen species (ROS) lead to lipid peroxidation of cell membrane, hepatocyte necrosis, and imbalance of antioxidant defense system, which directly affect the behavior of hepatic stellate cells and myofibroblasts, accompanied by the decrease in SOD level. Studies have shown that flavonoids from some plants can effectively control the effects of oxidative stress on liver (Mi et al., 2019). In the current study, the flavonoids of Wushan Shencha enhanced the expression of Cu/Zn‐SOD, Mn‐SOD, and CAT in mice with liver injury, thus might playing a role in preventing liver injury.

### 3.9 Nuclear factor kappa‐light‐chain‐enhancer of activated B cells (NF‐κB) and inhibitor (I)κB‐α of mRNA and protein expression in the liver tissue of mice

**Figure 5:**
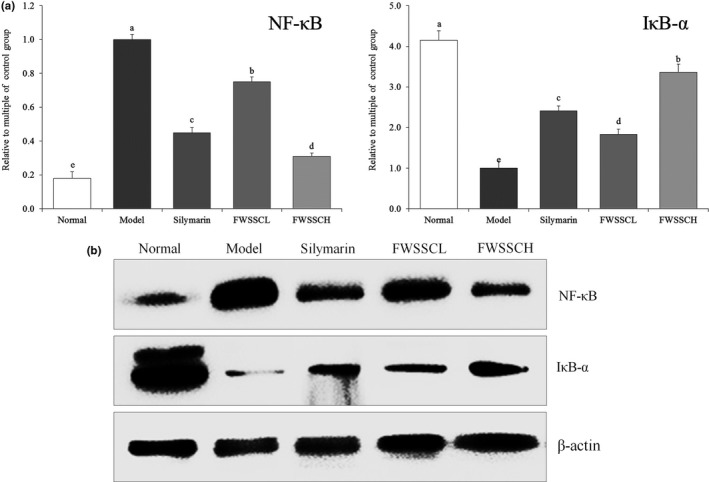
The mRNA (a) and protein (b) expression of NF‐κB and IκB‐α in the liver tissue of mice. Values presented are the mean ± standard deviation. ^a−e^Mean values with different letters in the bar are significantly different (*p* < .05) according to Duncan's new MRT. Silymarin group: 100 mg/kg b.w. silymarin treatment dose; FWSSCL group: 50 mg/kg b.w. Wushan Shencha flavonoids dose; FWSSCH group: 100 mg/kg b.w. Wushan Shencha flavonoids dose

Figure 5 shows that the mRNA and protein expression of NF‐κB in the liver tissue of normal mice was significantly lower than that of other groups (*p* < .05), while the expression of IκB‐α was significantly higher than that of other groups. The expression of NF‐κB and IκB‐α in the model group showed the opposite trend to that of the normal group. The expression of IκB‐α in the liver tissue of FWSSCH and silymarin mice was lower than that of normal mice, while the expression of NF‐κB was higher than that of normal mice.

Carbon tetrachloride decreases the stability of membranes, causes degeneration and necrosis of hepatocytes, leads to inflammation of the liver, and releases a large number of inflammatory factors. Inflammatory factor TNF‐α is mainly secreted by monocyte–macrophages and binds to TNF‐αR1 on the hepatocyte membrane, resulting in double‐stranded DNA becoming oligodeoxynucleotide fragments and apoptosis of stem cells (Morio et al., 2001). NF‐κB is a key factor in regulating inflammation. TNF‐α also aggravates inflammation by activating NF‐κB (Cai et al., 2018). NF‐κB is a central regulator of stress and inflammatory response. When the cell is at rest, NF‐κB binds to its inhibitory protein IκB and remains in the cell with inactive dimer. After inflammation, a large number of reactive oxygen species and oxygen free radicals are released. After stimulation of oxidation, the imbalance between oxidation and antioxidation tends to oxidize, leading to inflammatory infiltration of neutrophils, increased secretion of protease, and a large number of oxidative intermediates, such as leukotriene (LT), thromboxane A2 (TXA2), are proinflammatory mediators. Oxidative stress is a negative effect of free radicals in vivo, which is closely related to inflammation (Huang, Yang, Lamb, & Chen, 2017). When cells are stimulated by these signals, IκB kinase IKKs are phosphorylated, which lead to the ubiquitination and degradation of IκB. NF‐κB dissociates with IκB and exposes binding sites on Rel protein. NF‐κB is activated, binds to specific DNA sequences, induces the transcription and expression of related genes, and thus activates inflammatory factors and cytokines, leading to inflammation (Fu, Liu, Li, Jin, & Zhou, 2015; Bachmann, Waibler, Pleli, Pfeilschifter, & Mühl, 2017). In this study, flavonoids of Wushan Shencha might also regulate these genes, thereby further controlling oxidative stress and inhibiting liver injury.

### 3.10 Cyclooxygenase (COX)‐2, TNF‐α, and IL‐1β mRNA expression in the liver tissue of mice

**Figure 6:**
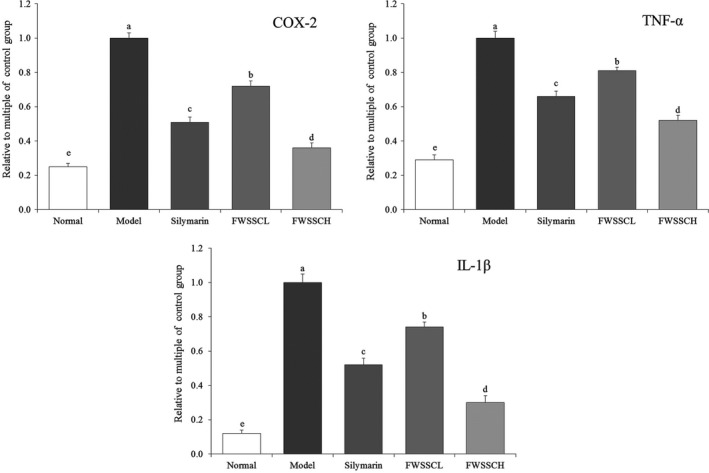
The mRNA expression of COX‐2, TNF‐α, and IL‐1β in the liver tissue of mice. Values presented are the mean ± standard deviation. ^a−e^Mean values with different letters in the bar are significantly different (*p* < .05) according to Duncan's new MRT. Silymarin group: 100 mg/kg b.w. silymarin treatment dose; FWSSCL group: 50 mg/kg b.w. Wushan Shencha flavonoids dose; FWSSCH group: 100 mg/kg b.w. Wushan Shencha flavonoids dose

Figure 6 shows that the mRNA expression of COX‐2, TNF‐α, and IL‐1β in liver tissue of normal mice exhibited the opposite trend with the expression of Cu/Zn‐SOD, Mn‐SOD, and CAT, which was significantly lower than that of the other groups (*p* < .05). The expression of COX‐2, TNF‐α, and IL‐1β in the liver tissue of the model mouse group was the strongest. The expression of COX‐2, TNF‐α, and IL‐1β in liver tissue induced by CCl_4_ was significantly affected (*p* < .05) by flavonoids from Wushan Shencha. The expression of COX‐2, TNF‐alpha, and IL‐1 β in liver tissue was decreased, which was similar to that in normal mice. There was no significant difference in the effect of the flavonoids in Wushan tea and silymarin at the same concentration (*p* > .05).

The inflammatory factor COX‐2 is not expressed in normal tissues. After the occurrence of liver injury, Kupffer cells are activated. COX‐2 is expressed and extensively synthesized, and the inflammatory damage of liver tissue is aggravated. After liver tissue damage, oxidative stress could lead to an imbalance of TNF‐α and IL‐1β inflammatory factors in vivo, and increase the amount of TNF‐α and IL‐1β in the liver (Bachmann et al., 2017). Overproduction of inflammatory cytokines can be used as a marker of liver injury, and inhibition of these inflammatory cytokines can help restore liver function. Overexpression of COX‐2 occurs when liver tissue is damaged. TNF‐α and IL‐1β are related to the pathogenesis of hepatotoxicity. Inflammatory cytokines TNF‐α and IL‐1β increased significantly within 24 hr of liver injury. Therefore, blocking the production of TNF‐α or IL‐1β may attenuate liver injury (Hu et al., 2019). In this study, the flavonoids of Wushan Shencha effectively enhanced the expression of IκB‐α and reduced the expression of COX‐2, TNF‐α, and IL‐1β in mice with liver injury. Activation of NF‐κB will lead to multiple verification processes, including oxidative stress induced by TNF‐α, including GSH‐Px, T‐SOD, ROS, XOD, and MDA abnormalities induced by TNF‐α. The regulation of TNF‐α by regulating the NF‐κB pathway may also play a role in inhibiting inflammation by enhancing the activity of antioxidant enzymes in vivo and reducing the harmful products produced by oxidative stress (Liu et al., 2015). Through the influence of these expressions, the effects of inflammation on the organism could be alleviated, which may be able to inhibit the abnormal expression of tissue caused by liver injury.

## 4 CONCLUSIONS

This study shows that the flavonoids of Wushan Shencha inhibit inflammation and oxidative stress in mice with liver injury. The flavonoids of Wushan Shencha can regulate liver function indexes and levels of inflammatory cytokines, and influence oxidative stress in liver tissues as well as verify the expression of related genes, so as to prevent liver injury induced by carbon tetrachloride in an all‐encompassing manner. It proves that the flavonoids of Wushan Shencha are beneficial for the liver. The bioactive components responsible for visceral protection have value and can be further developed and utilized. Based on basic animal experiments, this study has proved the role of the flavonoids in Wushan Shencha. In the future, in‐depth human trials will be needed to comprehensively verify the biological activity of flavonoids in Wushan Shencha.

## CONFLICT OF INTEREST

The authors of this manuscript state that they do not have conflict of interest to declare.

## ETHICAL REVIEW

This study has not any potential sources of conflict of interest. This study was conducted in accordance with the Declaration of Helsinki, and the protocol was approved by the Ethics Committee of Chongqing Collaborative Innovation Center for Functional Food, China.

## References

[fsn31243-bib-0001] Araújo Júnior, R. F. , Garcia, V. B. , Leitão, R. F. , Brito, G. A. , Miguel Ede, C. , Guedes, P. M. , & de Araújo, A. A. (2016). Carvedilol improves inflammatory response, oxidative stress and fibrosis in the alcohol‐induced liver injury in rats by regulating Kuppfer cells and hepatic stellate cells. PLoS ONE, 12, e0148868. 10.1371/journal.pone.0148868 PMC4758650 26891124

[fsn31243-bib-0002] Bachmann, M. , Waibler, Z. , Pleli, T. , Pfeilschifter, J. , & Mühl, H. (2017). Type I interferon supports inducible nitric oxide synthase in murine hepatoma cells and hepatocytes and during experimental acetaminophen‐induced liver damage. Frontiers in Immunology, 8, 890. 28824623 10.3389/fimmu.2017.00890 PMC5534483

[fsn31243-bib-0003] Bonthius, D. J. Jr , Winters, Z. , Karacay, B. , Bousquet, S. L. , & Bonthius, D. J. (2015). Importance of genetics in fetal alcohol effects: Null mutation of the nNOS gene worsens alcohol‐induced cerebellar neuronal losses and behavioral deficits. Neurotoxicology, 46, 60–72. 25511929 10.1016/j.neuro.2014.11.009 PMC4339445

[fsn31243-bib-0004] Cai, L. , Chen, W. N. , Li, R. , Hu, C. M. , Lei, C. , & Li, C. M. (2018). Therapeutic effect of acetazolamide, an aquaporin 1 inhibitor, on adjuvant‐induced arthritis in rats by inhibiting NF‐B signal pathway. Immunopharmacology and Immunotoxicology, 40, 117–125. 29303021 10.1080/08923973.2017.1417998

[fsn31243-bib-0005] Dong, D. , Zhang, S. , Yin, L. , Tang, X. , Xu, Y. , Han, X. , … Peng, J. (2013). Protective effects of the total saponins from Rosa laevigata Michx fruit against carbon tetrachloride‐induced acute liver injury in mice. Food and Chemical Toxicology, 62, 120–130. 23994094 10.1016/j.fct.2013.08.050

[fsn31243-bib-0006] Fu, X. J. , Liu, W. H. , Li, H. , Jin, C. H. , & Zhou, X. Q. (2015). Effects of Jianpi Butu prescription on the expression of NF‐κB and IκBα in rats with cerebral ischemia/reperfusion injury. Journal of Traditional Chinese Medicine University of Hunan, 35, 5–8.

[fsn31243-bib-0007] Huang, B. , Yang, X. D. , Lamb, A. , & Chen, L. F. (2017). Posttranslational modifications of NF‐kappaB: Another layer of regulation for NF‐kappaB signaling pathway. Cellular Signalling, 22, 1282–1290. 10.1016/j.cellsig.2010.03.017 PMC2893268 20363318

[fsn31243-bib-0008] Hu, J. N. , Xu, X. Y. , Li, W. , Wang, Y. M. , Liu, Y. , Wang, Z. , & Wang, Y. P. (2019). Ginsenoside Rk1 ameliorates paracetamol‐induced hepatotoxicity in mice through inhibition of inflammation, oxidative stress, nitrative stress and apoptosis. Journal of Ginseng Research, 43, 10–19. 30662289 10.1016/j.jgr.2017.07.003 PMC6323149

[fsn31243-bib-0009] Knekt, P. , Kumpulainen, J. , Järvinen, R. , Rissanen, H. , Heliövaara, M. , Reunanen, A. , … Aromaa, A. (2002). Flavonoid intake and risk of chronic diseases. American Journal of Clinical Nutrition, 76, 560–568. 12198000 10.1093/ajcn/76.3.560

[fsn31243-bib-0010] Knekt, P. , Isotupa, S. , Rissanen, H. , Heliövaara, M. , Järvinen, R. , Häkkinen, S. , … Reunanen, A. (2000). Quercetin intake and the incidence of cerebrovascular disease. European Journal of Clinical Nutrition, 54, 415–417. 10822289 10.1038/sj.ejcn.1600974

[fsn31243-bib-0011] Lee, C. H. , Park, S. W. , Kim, Y. S. , Kang, S. S. , Kim, J. A. , Lee, S. H. , & Lee, S. M. (2007). Protective mechanism of glycyrrhizin on acute liver injury induced by carbon tetrachloride in mice. Biological and Pharmaceutical Bulletin, 30, 1898–1904. 17917259 10.1248/bpb.30.1898

[fsn31243-bib-0012] Lee, M. H. , Hyun, D. H. , Jenner, P. , & Halliwell, B. (2001). Effect of proteasome inhibition on cellular oxidative damage, antioxidant defences and nitric oxide production. Journal of Neurochemistry, 78, 32–41. 11432971 10.1046/j.1471-4159.2001.00416.x

[fsn31243-bib-0013] Lettéron, P. , Labbe, G. , Degott, C. , Berson, A. , Fromenty, B. , Delaforge, M. , … Pessayre, D. (1990). Mechanism for the protective effects of silymarin against carbon tetrachloride‐induced lipid peroxidation and hepatotoxicity in mice. Evidence that silymarin acts both as an inhibitor of metabolic activation and as a chain‐breaking antioxidant. Biochemical Pharmacology, 39, 2027–2034. 2353942 10.1016/0006-2952(90)90625-u

[fsn31243-bib-0014] Liu, B. , Li, J. , Yi, R. , Mu, J. , Zhou, X. , & Zhao, X. (2019). Preventive effect of alkaloids from Lotus plumule on acute liver injury in mice. Foods, 8, 36. 10.3390/foods8010036 PMC6352077 30669459

[fsn31243-bib-0015] Liu, Y. , Zhu, L. , Liang, S. , Yao, S. , Li, R. , Liu, S. , … Wang, X. (2015). Galactose protects hepatocytes against TNF‐α‐induced apoptosis by promoting activation of the NF‐κB signaling pathway in acute liver failure. Laboratory Investigation, 95, 504–514. 25751739 10.1038/labinvest.2015.34

[fsn31243-bib-0016] Luckey, S. W. , & Petersen, D. R. (2001). Activation of Kupffer cells during the course of carbon tetrachloride‐induced liver injury and fibrosis in rats. Experimental and Molecular Pathology, 71, 226–240. 11733948 10.1006/exmp.2001.2399

[fsn31243-bib-0017] Manibusan, M. K. , Odin, M. , & Eastmond, D. A. (2007). Postulated carbon tetrachloride mode of action: A review. Journal of Environmental Science and Health Part C‐Environmental Carcinogenesis & Ecotoxicology Reviews, 25, 185–209. 10.1080/10590500701569398 17763046

[fsn31243-bib-0018] Mi, X. J. , Hou, J. G. , Jiang, S. , Liu, Z. , Tang, S. , Liu, X. X. , … Li, W. (2019). Maltol mitigates thioacetamide‐induced liver fibrosis through TGF‐β1‐mediated activation of PI3K/Akt signaling pathway. Journal of Agricultural and Food Chemistry, 67, 1392–1401. 30644744 10.1021/acs.jafc.8b05943

[fsn31243-bib-0019] Morio, L. A. , Chiu, H. , Sprowles, K. A. , Zhou, P. , Heck, D. E. , Gordon, M. K. , & Laskin, D. L. (2001). Distinct roles of tumor necrosis factor‐alpha and nitric oxide in acute liver injury induced by carbon tetrachloride in mice. Toxicology and Applied Pharmacology, 172, 44–51. 11264022 10.1006/taap.2000.9133

[fsn31243-bib-0020] Ohta, Y. , Kongo, M. , Sasaki, E. , Nishida, K. , & Ishiguro, I. (1999). Preventive effect of melatonin on the progression of carbon tetrachloride‐induced acute liver injury in rats. Advances in Experimental Medicine and Biology, 467, 327–332. 10721073 10.1007/978-1-4615-4709-9_42

[fsn31243-bib-0021] Pramyothin, P. , Janthasoot, W. , Pongnimitprasert, N. , Phrukudom, S. , & Ruangrungsi, N. (2004). Hepatotoxic effect of (+)usnic acid from Usnea siamensis Wainio in rats, isolated rat hepatocytes and isolated rat liver mitochondria. Journal of Ethnopharmacology, 90, 381–387. 15013205 10.1016/j.jep.2003.10.019

[fsn31243-bib-0022] Pan, Y. , Long, X. , Yi, R. , & Zhao, X. (2018). Polyphenols in Liubao tea can prevent CCl_4_‐induced hepatic damage in mice through its antioxidant capacities. Nutrients, 10, 1280. 10.3390/nu10091280 PMC6163653 30201943

[fsn31243-bib-0023] Qian, Y. , Zhang, J. , Fu, X. , Yi, R. , Sun, P. , Zou, M. , … Zhao, X. (2018). Preventive effect of raw Liubao tea polyphenols on mouse gastric injuries induced by HCl/ethanol via anti‐oxidative stress. Molecules, 23, 2848. 10.3390/molecules23112848 PMC6278666 30388863

[fsn31243-bib-0024] Sharangi, A. B. (2009). Medicinal and therapeutic potentialities of tea (*Camellia sinensis* L.) ‐ A review. Food Research International, 42, 529–535.

[fsn31243-bib-0025] Shim, J. Y. , Kim, M. H. , Kim, H. D. , Ahn, J. Y. , Yun, Y. S. , & Song, J. Y. (2009). Protective action of the immunomodulator ginsan against carbon tetrachloride‐induced liver injury via control of oxidative stress and the inflammatory response. Toxicology and Applied Pharmacology, 242, 318–325. 19913046 10.1016/j.taap.2009.11.005

[fsn31243-bib-0026] Sipes, I. G. , el Sisi, A. E. , Sim, W. W. , Mobley, S. A. , & Earnest, D. L. (1991). Reactive oxygen species in the progression of CCl_4_‐induced liver injury. Advances in Experimental Medicine and Biology, 283, 489–497. 2069020 10.1007/978-1-4684-5877-0_65

[fsn31243-bib-0027] Trueba, G. P. , Sánchez, G. M. , & Giuliani, A. (2004). Oxygen free radical and antioxidant defense mechanism in cancer. Frontiers in Bioscience, 9, 2029–2044. 15353268 10.2741/1335

[fsn31243-bib-0028] van Acker, S. A. , van den Berg, D. J. , Tromp, M. N. , Griffioen, D. H. , van Bennekom, W. P. , van der Vijgh, W. J. , & Bast, A. (1996). Structural aspects of antioxidant activity of flavonoids. Free Radical Biology and Medicine, 20, 331–342. 8720903 10.1016/0891-5849(95)02047-0

[fsn31243-bib-0029] Wang, L. , Cheng, D. , Wang, H. , Di, L. , Zhou, X. , Xu, T. , … Liu, Y. (2009). The hepatoprotective and antifibrotic effects of *Saururus chinensis* against carbon tetrachloride induced hepatic fibrosis in rats. Journal of Ethnopharmacology, 126, 487–491. 19761824 10.1016/j.jep.2009.09.009

[fsn31243-bib-0030] Wang, R. , Yang, Z. , Zhang, J. , Mu, J. , Zhou, X. , & Zhao, X. (2019). Liver injury induced by carbon tetrachloride in mice is prevented by the antioxidant capacity of Anji white tea polyphenols. Antioxidants, 8, 64. 10.3390/antiox8030064 PMC6466528 30875793

[fsn31243-bib-0031] Weber, L. W. , Boll, M. , & Stampfl, A. (2003). Hepatotoxicity and mechanism of action of haloalkanes: Carbon tetrachloride as a toxicological model. Critical Reviews in Toxicology, 33, 105–136. 12708612 10.1080/713611034

[fsn31243-bib-0032] Xie, J. , Wang, W. , Dong, C. , Huang, L. , Wang, H. , Li, C. , … Xie, M. (2018). Protective effect of flavonoids from *Cyclocarya paliurus* leaves against carbon tetrachloride‐induced acute liver injury in mice. Food and Chemical Toxicology, 119, 392–399. 29337229 10.1016/j.fct.2018.01.016

[fsn31243-bib-0033] Yi, Q. Y. , Wu, X. X. , Tan, Y. , Li, X. , & Zhao, X. (2014). Influence of Wushan Shencha aqueous extract on small intestine peristalsis in Kunming mice. Journal of ZhongKai University of Agriculture and Technology, 2014, 57–59.

[fsn31243-bib-0034] Yuan, L. P. , Chen, F. H. , Ling, L. , Bo, H. , Chen, Z. W. , Li, F. , … Xia, L. J. (2008). Protective effects of total flavonoids of *Bidens bipinnata* L. against carbon tetrachloride‐induced liver fibrosis in rats. Journal of Pharmacy and Pharmacology, 60, 1393–1402. 18812033 10.1211/jpp/60.10.0016

[fsn31243-bib-0035] Zhang, J. , Zhou, X. , Chen, B. , Long, X. , Mu, J. , Pan, Y. , … Yang, Z. (2018). Preventive effect of *Lactobacillus plantarum* CQPC10 on activated carbon induced constipation in Institute of Cancer Research (ICR) mice. Applied Sciences, 8, 1498.

[fsn31243-bib-0036] Zhang, S. , Lu, B. , Han, X. , Xu, L. , Qi, Y. , Yin, L. , … Peng, J. (2012). Protection of the flavonoid fraction from Rosa laevigata Michx fruit against carbon tetrachloride‐induced acute liver injury in mice. Food and Chemical Toxicology, 55, 60–69. 23279844 10.1016/j.fct.2012.12.041

[fsn31243-bib-0037] Zhao, X. , Qian, Y. , Li, G. J. , & Tan, J. (2015). Preventive effects of the polysaccharide of Larimichthys crocea swim bladder on carbon tetrachloride (CCl_4_)‐induced hepatic damage. Chinese Journal of Natural Medicines, 13, 521–528. 26233842 10.1016/S1875-5364(15)30046-7

